# Axial Torsion of a Necrotic Giant Meckel’s Diverticulum With Secondary Peritonitis in a 7‐Year‐Old: A Case Report

**DOI:** 10.1155/cris/9903442

**Published:** 2026-04-28

**Authors:** Maria Daniela Trăilescu, Adrian Ionel Pavel, Adrian Silviu Crișan, Carmen Nicoleta Cindea, Henry Ifeanyi Osakwe, Georgiana Maria Boștinaru

**Affiliations:** ^1^ “Vasile Goldiș” Western University of Arad, Faculty of Medicine, Strada Liviu Rebreanu 86, Arad, Romania; ^2^ County Emergency Hospital Arad, Calea Victoriei 1–3, Arad, Romania; ^3^ “Victor Babeș” University of Medicine and Pharmacy Timișoara, Piata Eftimie Murgu No. 2, Timișoara, 300041, Romania, umft.ro

**Keywords:** abdominal pain, axial torsion, laparotomy, laparotomy, Meckel’s diverticulum, pediatric acute abdomen, small bowel obstruction

## Abstract

Axial torsion with subsequent gangrene of Meckel’s diverticulum represents an exceptionally rare cause of small bowel obstruction, one of the rarest causes of small bowel obstruction. Meckel’s diverticulum is the most common congenital anomaly of the gastrointestinal tract, and it is often discovered incidentally during abdominal surgery, particularly in pediatric patients. Approximately 4% of individuals with Meckel’s diverticulum develop complications, including bleeding, obstruction, inflammation, intussusception, or perforation, with torsion being the rarest. Despite its rarity, symptomatic Meckel’s diverticulum poses serious diagnostic and therapeutic challenges due to its nonspecific clinical presentation, which may mimic other intra‐abdominal pathologies. Consequently, preoperative diagnosis is difficult, and delayed management may lead to life‐threatening complications. We report a case of axial gangrenous torsion of a giant Meckel’s diverticulum in a child without a history of previous abdominal surgery. In such cases, prompt decision‐making for exploratory laparotomy or laparoscopy is crucial to achieving a favorable outcome. We report a case of axial torsion with gangrene of a giant Meckel’s diverticulum in a 7‐year‐old boy with no prior history of abdominal surgery. This case highlights the importance of early recognition and timely operative management in atypical presentations of acute abdomen in children.

## 1. Introduction

Meckel’s diverticulum, first described by Johann Friedrich Meckel in 1809, arises from incomplete obliteration of the vitelline (omphalomesenteric) duct between the 5th and 7th weeks of gestation [1–6]. Depending on the extent of persistence, this anomaly may present as an umbilical fistula, sinus, or Meckel’s diverticulum [7]. Histologically, Meckel’s diverticulum is a true diverticulum containing all layers of the intestinal wall and may harbor ectopic mucosa‐most commonly gastric or pancreatic‐in to 50% of cases [7]. It is typically located on the antimesenteric border of the ileum, within 90 cm of the ileocecal valve in about 90% of patients [4]. Although most cases remain asymptomatic, complications may occur, particularly in males and usually present with gastrointestinal bleeding secondary to peptic ulceration of adjacent ileal mucosa associated with ectopic gastric tissue [8]. These include gastrointestinal bleeding, obstruction, inflammation, intussusception, and rarely torsion. The average length of a diverticulum measures 2.9 cm and a width of about 1.9 cm, though giant diverticula exceeding 5 cm have been reported, with sizes reaching up to 16 cm in length [4, 9]. Axial torsion, defined as rotation around the diverticulum’s vascular pedicle leading to ischemia and gangrene, is an exceedingly rare entity, especially in the pediatric population [4]. Preoperative diagnosis is challenging due to nonspecific clinical findings and overlap with more common conditions such as appendicitis [10]. This report describes a rare case of axial torsion of a giant Meckel’s diverticulum in a child and discusses the diagnostic and therapeutic considerations, compares our findings with previous literature findings with about 20 previously reported cases in children [11] and about 20 more cases in adults.

## 2. Case Presentation

**Figure 1:**
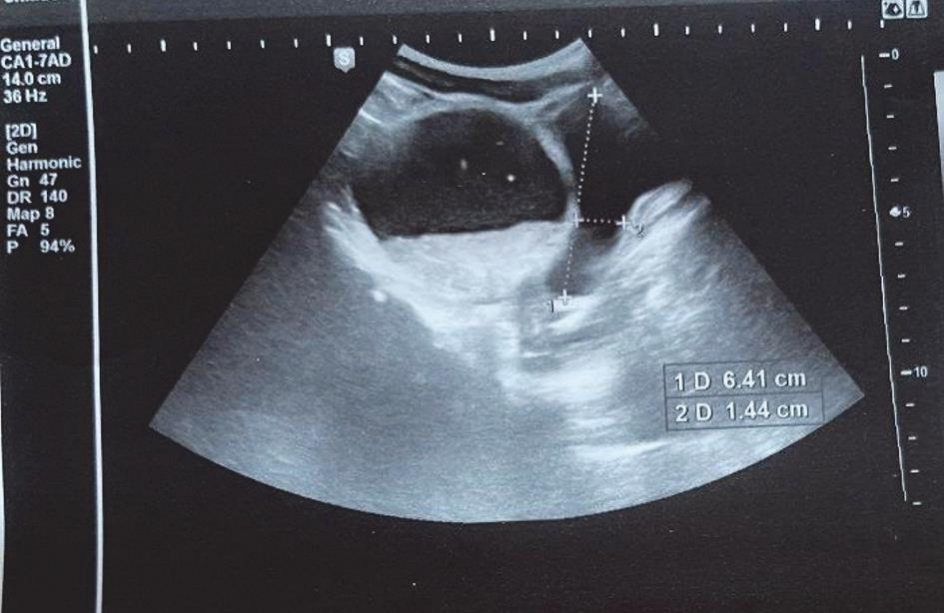
Abdominal ultrasonography demonstrating free intraperitoneal fluid and a large para‐vesical cystic mass measuring 12.5 cm × 7 cm.

**Figure 2:**
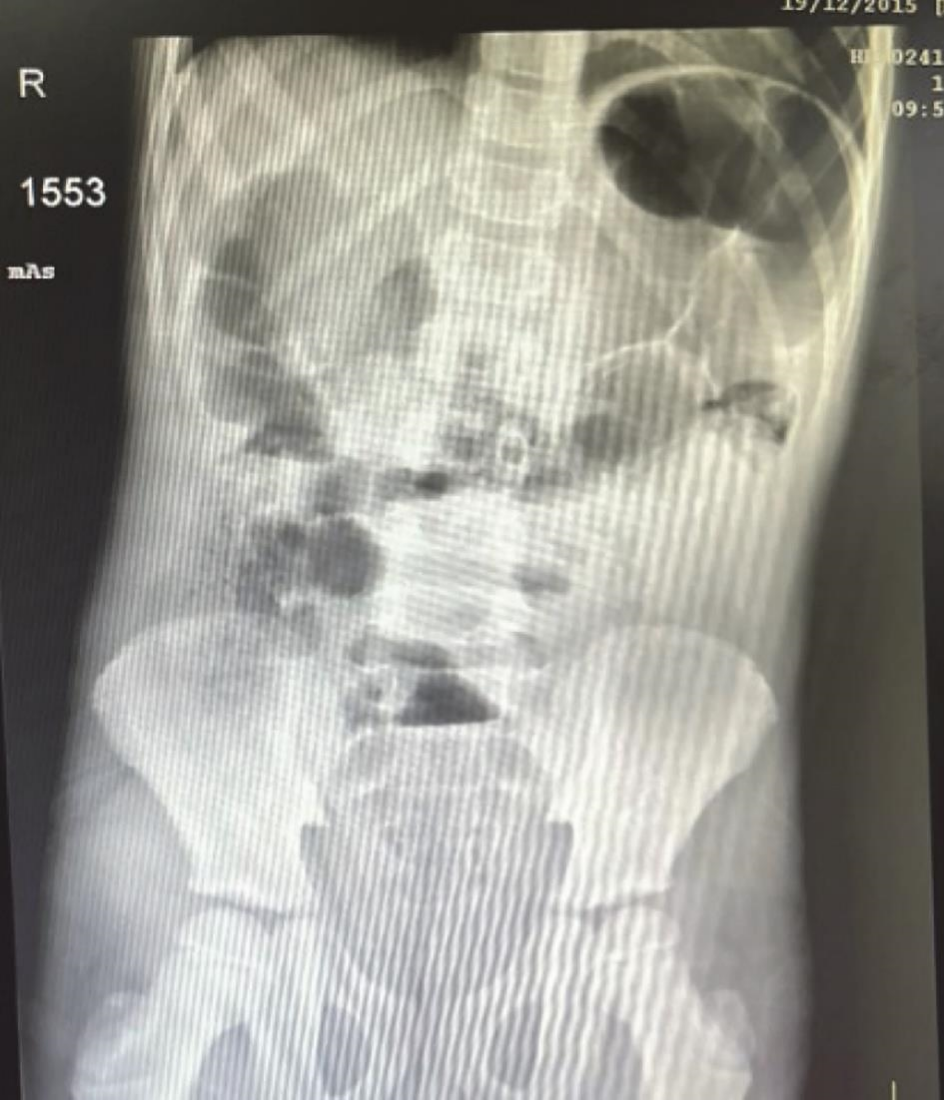
Abdominal X‐ray showing multiple air–fluid levels consistent with small bowel obstruction.

**Figure 3:**
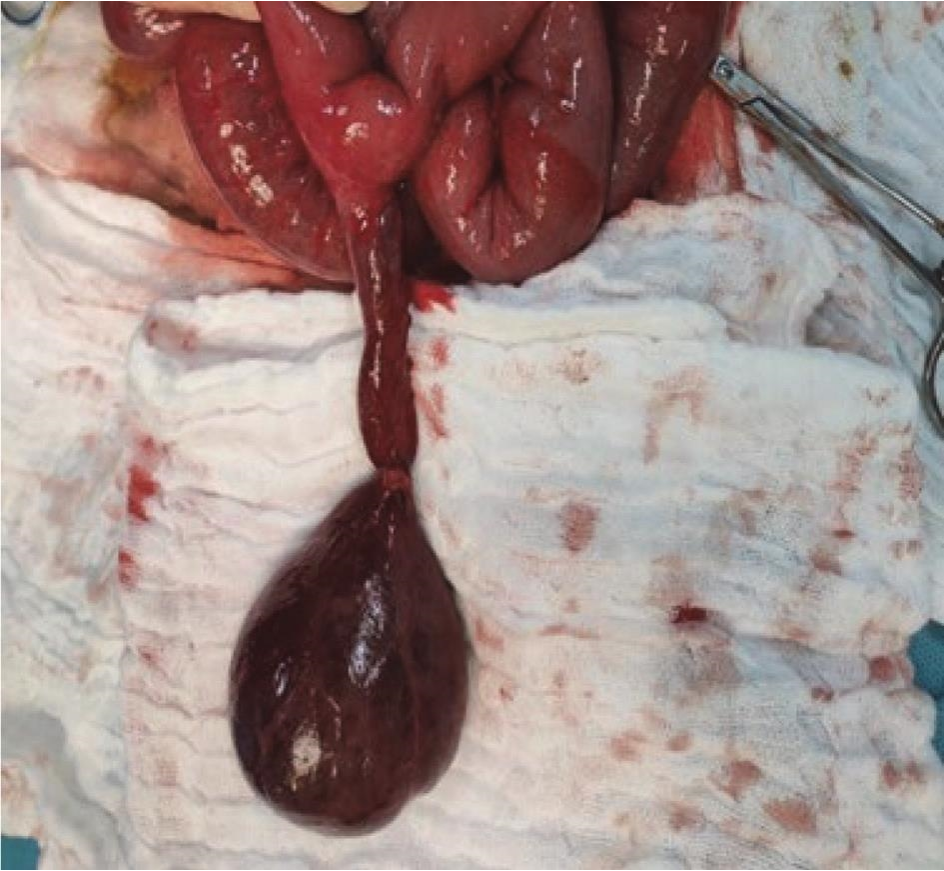
Intraoperative image demonstrating 720° axial torsion of a giant Meckel’s diverticulum causing ischemia, small bowel obstruction, and necrosis.

**Figure 4:**
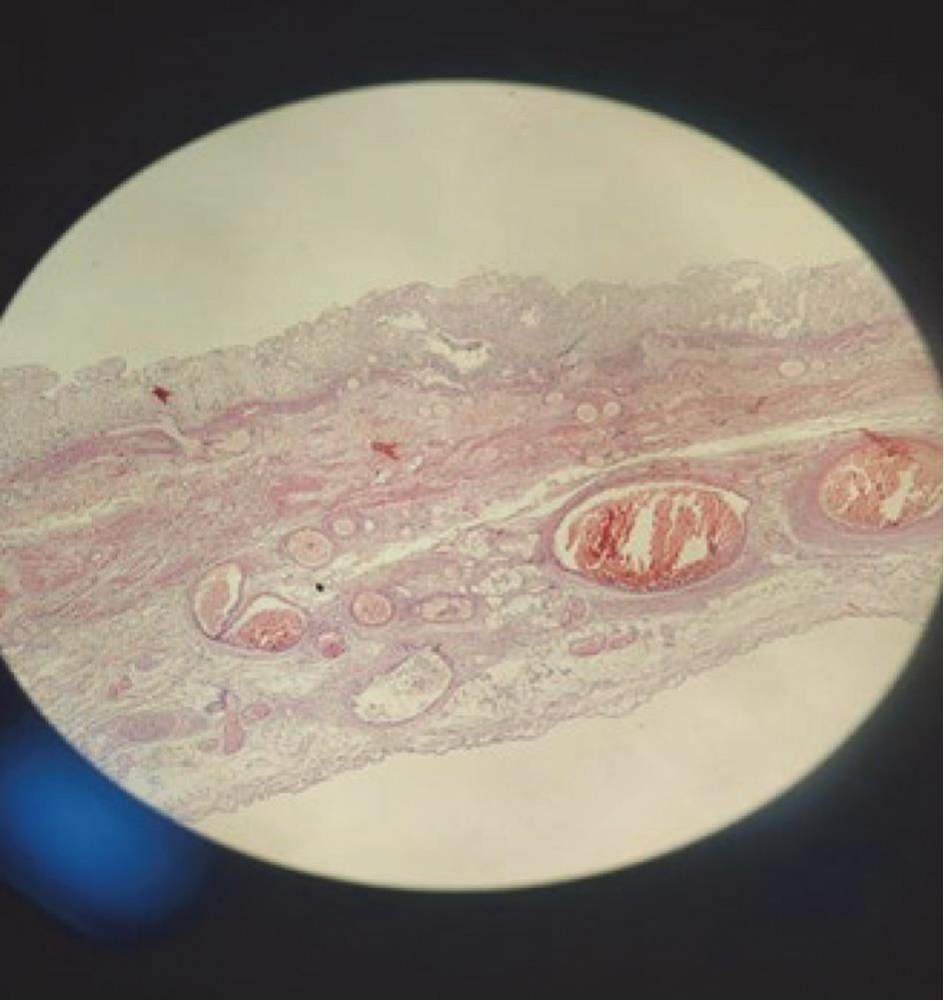
Histopathological examination of the resected Meckel’s diverticulum. Histologic aspect—Meckel diverticulum showing thin epithelial mucosal wall with vascular congestion, fibro‐hematic thrombosis, moderate inflammatory infiltration, and ischemic necrosis without ectopic tissue (staining method: hematoxylin and eosin stain, magnification: 40×, scale bar: 1 mm).

A 7‐year‐old boy presented to the emergency department with a 2‐day history of crampy abdominal pain, nausea, and vomiting. His mother also reported the absence of stool passage. The patient was afebrile. On physical examination, the child appeared fatigued, with tenderness localized predominantly in the right lower quadrant. There was no guarding or signs of peritonitis on presentation. Digital rectal examination was unremarkable. Initial laboratory investigations showed: normal leukocyte count (8540/μL), normal CRP (0.60 mg/L), and normal renal, hepatic, and hemoglobin values. Abdominal radiography demonstrated dilated small bowel loops. Patient was initially managed conservatively with intravenous fluids, pantoprazole (20 mg), drotaverine (40 mg), paracetamol (10 mg/mL), and ceftriaxone (1 g). After 12 h, the patient’s condition deteriorated clinically, with worsening pain and abdominal distension. Laboratory findings showed: WBC (23,780/μL), CRP 8.27 mg/L, and Neutrophilia (91.8%). Procalcitonin was within normal range. Ultrasound showed free peritoneal fluid and a large para‐vesical cystic mass measuring 12.5 cm × 7 cm (Figure 1). Repeat imaging showed multiple air–fluid levels, suggestive of bowel obstruction (Figure 2). An emergency subumbilical midline laparotomy identified ~200 mL of intraperitoneal fluid and a giant, gangrenous Meckel’s diverticulum twisted 720° around its vascular pedicle (12.5 cm × 7.0 cm), located about 30 cm from the ileocecal valve (Figure 3). The omphalomesenteric duct was intact. The diverticulum was detorsed, and wedge resection was performed, followed by V‐shaped ileal resection with enteroplasty. An appendectomy was also carried out. The postoperative outcome was uneventful, and the patient was discharged on postoperative day 6. Follow‐up at 4 months showed no complications. Histopathological examination showed: thin epithelial mucosal lining with vascular congestion, fibro‐hematic thrombosis, polymorphous inflammatory infiltration, edema, and extensive ischemic necrosis, absence of ectopic tissue or neoplasia (Figure 4). Resection margins were free of pathology.

## 3. Discussion

Meckel’s diverticulum results from incomplete obliteration of the vitelline duct [5]. Axial torsion occurs when the diverticulum rotates around its vascular pedicle, causing a serious complication leading to ischemia and gangrene [6]. Several predisposing factors have been described, including: persistency of a fibrous vitelline band attaching the diverticulum to the umbilicus [7], increased diverticular mobility, narrow base with a long meso‐diverticular pedicle, and the presence of intradiverticular masses [5, 12–14]. Though the exact mechanism of torsion remains unclear. The clinical presentation is often nonspecific and may mimic acute appendicitis, contributing to diagnostic delay. In this case, the initial presentation was not suggestive of an acute abdomen and laboratory findings were unremarkable, further complicating early diagnosis. Initial presentation mimicked a nonacute abdomen, with nonspecific clinical and paraclinical findings, contributing to diagnostic uncertainty. Imaging plays a critical role, although findings are often indirect. Ultrasound is particularly useful in pediatric patients due to the absence of radiation, while computer tomography may provide more specific diagnostic clues. The progression from mild symptoms to gangrene suggests a gradual evolving torsion mechanism. Early imaging and a higher index of suspicion might have facilitated earlier intervention. The choice of surgical approach depends on intraoperative findings and patient condition. Although laparoscopy is increasingly utilized, laparotomy remains preferable in cases involving large diverticular, suspected necrosis and hemodynamic instability. Given the large size of the diverticulum (12.5 cm × 7.0 cm), exploratory laparotomy was preferred over laparoscopy. There is no strict, universally accepted size threshold of a Meckel’s diverticulum that *by itself* mandates laparotomy in cases of torsion. The decision is primarily context‐driven, not based on a single measurement. However, literature findings and surgical practice do suggest practical size‐related considerations: above 5 cm diameter [15], published studies suggest that laparoscopic approaches in similar cases may occasionally necessitate conversion to open surgery to ensure patient safety. Segmental resection is recommended for wide‐based diverticular or when ectopic tissue is suspected, whereas wedge resection is appropriate for narrow‐based lesions. Ultimately, prompt recognition of an acute abdomen and immediate surgical exploration is essential to prevent necrosis and peritonitis. Appendectomy is often performed during surgery for Meckel’s diverticulum as a complementary procedure, even when the appendix appears macroscopically normal, for several important clinical reasons: prevents future diagnostic confusion, plays a prophylactic role and eliminates the risk of subsequent appendicitis.

## 4. Conclusion

Axial torsion of Meckel’s diverticulum is rare but an important differential diagnosis in pediatric patients presenting with acute abdomen or bowel obstruction, particularly in the absence of prior abdominal surgery. In the absence of early symptoms and prompt imaging, a high index of suspicion is crucial for timely diagnosis. Early recognition, appropriate imaging, and prompt surgical intervention are essential to prevent severe complications such as gangrene and peritonitis.

## Author Contributions

**Maria Daniela Trăilescu:** supervision (lead), project administration (equal), writing – review and editing (equal). **Henry Ifeanyi Osakwe:** conceptualization (lead), methodology (equal), writing – original draft preparation (lead), writing – review and editing (equal). **Adrian Ionel Pavel:** data curation (lead), investigation (equal). **Adrian Silviu Crișan:** formal analysis (equal), visualization (equal). **Carmen Nicoleta Cindea:** data analysis (equal), resources (supporting). **Georgiana Maria Boștinaru:** data curation (equal), validation (supporting), writing – review and editing (supporting).

## Funding

No financial support was received for this study.

## Disclosure

All authors read and approved the final manuscript.

## Ethics Statement

This article does not contain any studies with human participants or animals performed by the authors. The authors declare no sources of funding. The work complies with the CARE case report guidelines.

## Consent

Written informed consent was obtained from the patient’s parents for publication of this case report and accompanying images.

## Conflicts of Interest

The authors declare no conflicts of interest.

## Data Availability

Data sharing is not applicable to this article as no datasets were generated or analyzed during the current study.

## References

[1] Yamaguchi M. , Takeuchi S. , and Awazu S. , Meckel’s Diverticulum: Investigation of 600 Patients in Japanese Literature, The American Journal of Surgery. (1978) 136, no. 2, 247–249, 10.1016/0002-9610(78)90238-6, 2-s2.0-0018185307. 308325

[2] Sagar J. , Kumar V. , and Shah D. K. , Meckel’s Diverticulum: A Systematic Review, Journal of the Royal Society of Medicine. (2006) 99, no. 10, 501–505, 10.1177/014107680609901011. 17021300 PMC1592061

[3] Prall R. T. , Bannon M. P. , and Bharucha A. E. , Meckel’s Diverticulum Causing Intestinal Obstruction, The American Journal of Gastroenterology. (2001) 96, no. 12, 3426–3427, 10.1111/j.1572-0241.2001.05344.x. 11774961

[4] Limas C. , Seretis K. , Soultanidis C. , and Anagnostoulis S. , Axial Torsion and Gangrene of a Giant Meckel’s Diverticulum, Journal of gastrointestinal and liver diseases. (2006) 15, no. 1, 67–68. 16680236

[5] Guss D. A. and Hoyt D. B. , Axial Volvulus of Meckel’s Diverticulum: A Rare Cause of Acute Abdominal Pain, Annals of Emergency Medicine. (1987) 16, no. 7, 811–812, 10.1016/S0196-0644(87)80583-8, 2-s2.0-0023250607. 3592339

[6] Moore G. P. and Burkle F. M. , Isolated Axial Volvulus of a Meckel’s Diverticulum, The American Journal of Emergency Medicine. (1988) 6, no. 2, 137–142, 10.1016/0735-6757(88)90052-6, 2-s2.0-0023929159. 3281683

[7] Tan Y.-M. and Zheng Z.-X. , Recurrent Torsion of a Giant Meckel’s Diverticulum, Digestive Diseases and Sciences. (2005) 50, no. 7, 1285–1287, 10.1007/s10620-005-2774-7, 2-s2.0-23044499163. 16047474

[8] Gagnier J. , Kienle G. , Altman D. G. , Moher D. , Sox H. , and Riley D. S. , The CARE Guidelines: Consensus-Based Clinical Case Report Guideline Development, Journal of Clinical Epidemiology. (2014) 67, no. 1, 46–51, 10.1016/j.jclinepi.2013.08.003, 2-s2.0-84888308413. 24035173

[9] Prasad T. R. , Chui C. H. , and Jacobsen A. S. , Laparoscopic Resection of an Axially Torted Meckel’s Diverticulum in a 13-Year-Old, Journal of Laparoendoscopic & Advanced Surgical Techniques. (2006) 16, no. 4, 425–427, 10.1089/lap.2006.16.425, 2-s2.0-33751565995. 16968199

[10] Mackey W. C. and Dineen P. , A Fifty-Year Experience With Meckel’s Diverticulum, Surgery Gynecology & Obstetrics. (1983) 156, 56–64. 6600203

[11] Hu Y. , Jia L. , Wang Y. , Xin Y. , and Wang X. , Sonographic Features of Axial Torsion of Meckel’s Diverticulum in 12 Pediatric Patients: A Retrospective Analysis and Literature Review, BMC Pediatrics. (2025) 25, no. 1, 10.1186/s12887-025-05826-y, 564. 40696289 PMC12285082

[12] Toshihiko W. and Hirofumi O. , Two Cases of Meckel’s Diverticulum Torsion, The Japanese Journal of Gastroenterological Surgery. (2002) 35, no. 2, 180–183, 10.5833/jjgs.35.180, 2-s2.0-0036190071.

[13] Almagro U. A. and Erickson L.Jr, Fibroma in Meckel’s Diverticulum: A Case Associated With Axial and Ileal Volvulus, The American Journal of Gastroenterology. (1982) 77, 477–480. 7091137

[14] Malik A. A. , Wani K. A. , and Khaja A. R. , Meckel’s Diverticulum Revisited, Saudi Journal of Gastroenterology. (2010) 16, no. 1, 3–7, 10.4103/1319-3767.58760, 2-s2.0-75649100573. 20065566 PMC3023098

[15] Erol V. , Yoldaş T. , Cin S. , Çalışkan C. , Akgün E. , and Korkut M. , Complicated Meckel’s Diverticulum and Therapeutic Management, Ulus Travma Acil Cerrahi Derg. (2013) 29, no. 2, 63–66, 10.5152/UCD.2013.36, 2-s2.0-84882371855. PMC4379835 25931848

